# JMJD8 is a positive regulator of TNF-induced NF-κB signaling

**DOI:** 10.1038/srep34125

**Published:** 2016-09-27

**Authors:** Kok Siong Yeo, Ming Cheang Tan, Wan Ying Wong, Sheng Wei Loh, Yi Lyn Lam, Chin Leng Tan, Yat-Yuen Lim, Chee-Kwee Ea

**Affiliations:** 1 Institute of Biological Sciences, Faculty of Science, University of Malaya, 50603 Kuala Lumpur, Malaysia

## Abstract

TNF-induced signaling mediates pleiotropic biological consequences including inflammation, immunity, cell proliferation and apoptosis. Misregulation of TNF signaling has been attributed as a major cause of chronic inflammatory diseases and cancer. Jumonji domain-containing protein 8 (JMJD8) belongs to the JmjC family. However, only part of the family members has been described as hydroxylase enzymes that function as histone demethylases. Here, we report that JMJD8 positively regulates TNF-induced NF-κB signaling. Silencing the expression of JMJD8 using RNA interference (RNAi) greatly suppresses TNF-induced expression of several NF-κB-dependent genes. Furthermore, knockdown of JMJD8 expression reduces RIP ubiquitination, IKK kinase activity, delays IκBα degradation and subsequently blocks nuclear translocation of p65. In addition, JMJD8 deficiency enhances TNF-induced apoptosis. Taken together, these findings indicate that JMJD8 functions as a positive regulator of TNF-induced NF-κB signaling.

The tumor necrosis factor (TNF) superfamily consists of 19 ligands and 29 receptors with diverse physiological functions^1^. Among the family members, TNFα and TNFR1 are the most well characterized ligand and receptor. As a pleiotropic pro-inflammation cytokine, TNFα regulates many biological processes namely inflammation, immunity, cell proliferation and apoptosis^2^,^3^. Stimulating cells with TNFα activates NF-κB and MAP kinases including ERK, p38 and JNK. In the TNFR1 signaling, engagement of TNFα with TNFR1 leads to the recruitment of the TNFR1-associated death domain (TRADD) protein. TRADD subsequently serves as a platform for the recruitment of FAS-associated death domain (FADD) protein, TNF receptor-associated factor 2 (TRAF2) protein and the death domain kinase RIP1. While association of FADD with TRADD triggers the apoptosis program, binding of TRAF2 and RIP1 to TRADD activates NF-κB and JNK^4^,^5^.

NF-κB consists of five members including p65 (RelA), RelB, cRel, p50/p105 (NF-κB1) and p52/p100 (NF-κB2), which can form either homo- or heterodimers^6^,^7^. In resting cells, NF-κB is sequestered in the cytoplasm and bound to its inhibitor, IκB family members. Upon stimulation, IκB is phosphorylated by an upstream kinase complex consists of IKKα, IKKβ and NEMO which leads to its degradation via the ubiquitin-proteasome pathway. Free NF-κB is then translocated into the nucleus to activate its target genes^6^,^7^,^8^. Although the activity of NF-κB is primarily regulated by its translocation into the nucleus, post-translational modifications of the NF-κB protein have distinct functional significances in regulating the activity of NF-κB protein. Recently, many post-translational modifications such as acetylation, phosphorylation, ubiquitination and methylation of the NF-κB members have been shown to regulate the NF-κB activities^9^,^10^,^11^. For example, previous studies showed that methylation of p65 at lysine 37 (K37) by a methytransferase, SET9 modulates its function^10^, while acetylation of p65 at K218 and K221 inhibits IκB binding and enhances DNA binding^12^, and acetylation of p65 at K122 and K123 inhibits its transcriptional activation activity^13^. These post-translational modifications are reversible. To date, only one group has reported that p65 is regulated by demethylase, namely FBXL11^14^,^15^. However, it is unclear whether NF-κB activity is also regulated by other demethylases.

Jumonji domain-containing (JMJD) proteins were first reported by Takeuchi’s group^16^. There are more than 30 protein members identified in mammals that contain Jumonji C (JmjC) domain^17^. Most of the JmjC domain-containing proteins are hydroxylase enzymes that function as demethylases^18^. Many proteins in this family have been shown to be involved in cell development, differentiation and proliferation through regulating various signaling pathways. On the other hand, deregulation of JMJD proteins can lead to various human malignancies^16^,^19^. For example, JMJD2C (also known as GASC1) is upregulated in squamous cell carcinoma^20^ and it regulates cell proliferation^21^. JmjC family members classified as histone demethylases usually contain known histone-binding domains such as PHD and Tudor domains^19^. However, to date, only part of the family members function as histone demethylase^19^ and the function of many JMJD proteins are not known.

Jumonji domain-containing protein 8 (JMJD8) is a JmjC domain-only protein that contains a JmjC domain at 74–269 amino acid residues with no other recognizable protein domains. Here, we examine the role of JMJD8 in TNF signaling and demonstrate that JMJD8 is a positive regulator for TNF-induced NF-κB signaling.

## Results

### JMJD8 is required for TNF-induced NF-κB-dependent gene expression

**Figure 1:**
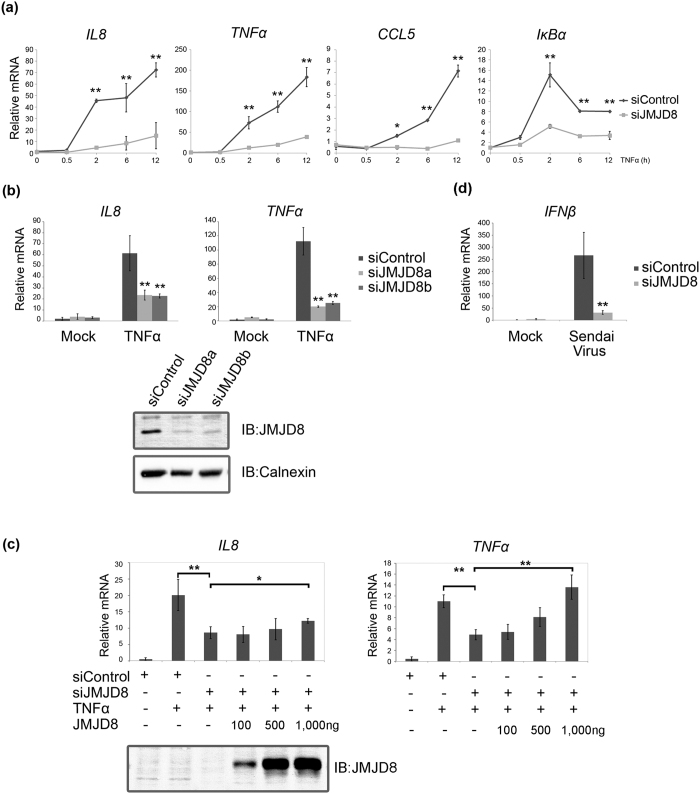
JMJD8 positively regulates NF-κB. (**a**) HEK293T cells transfected with control and JMJD8 targeting siRNA oligos were treated with and without 10 ng/ml of TNFα for 0, 0.5, 2, 6 and 12 hours. The expression of *TNFα*, *IL8*, *CCL5* and *IκBα* were measured by RT-qPCR (n = 4). (**b**) HEK293T cells transfected with control, JMJD8a and JMJD8b siRNA oligos were treated with and without 10 ng/ml of TNFα for 2 hours, the expression of *TNFα* and *IL8* were measured by RT-qPCR. The knockdown expression of JMJD8 by each siRNA oligo was verified by immunobloting with a JMJD8 specific antibody (n = 4). (**c**) JMJD8 knockdown HEK293T cells reconstituted with JMJD8 were treated with TNFα for 2 hours and the expression of *TNFα* and *IL8* were measured by RT-qPCR. The transient expression of ectopic JMJD8 was verified by immunobloting with a JMJD8 specific antibody (n = 4). (**d**) Control and JMJD8 knockdown HEK293T cells were infected with Sendai virus (150 HAU/ml) and the levels of *IFNβ* were measured by RT-qPCR (n = 4). Data represent the means ± SD. (*p > 0.05, **p > 0.01). Full-length blots are presented in Supplementary Fig. S3.

Our previous finding that methylation of p65 protein regulates its transcriptional activity^10^ prompted us to evaluate whether demethylases are also involved in TNF-induced NF-κB signaling. We did RNAi screening of a group of Jumonji domain-containing proteins and found that the JMJD8, a JmjC domain-only protein may be involved in regulating TNF-induced NF-κB signaling (Data not shown). To verify our observation, we compared the TNF-induced transcription kinetics of a few well-known NF-κB-dependent genes between control and JMJD8 knockdown HEK293T cells. As shown in Fig. 1a, the TNF-induced NF-κB transcriptional activity was almost completely abrogated in JMJD8 knockdown cells compared to the control cells. The effect of JMJD8 knockdown on TNF-induced NF-κB signaling was further supported by a NF-κB luciferase reporter assay (see Supplementary Fig. S1a).

To ensure that the NF-κB activation defect observed in the JMJD8 knockdown cells is not due to an off-target effect, we tested a second siRNA oligo that targets an alternative site of the JMJD8 transcript. The knockdown of JMJD8 by each siRNA oligo was verified by immunoblotting with a JMJD8 specific antibody (Fig. 1b, lower panel). In line with the previous observation, transfection of both siRNA oligos specific for JMJD8 into HEK293T cells not only resulted in a decrease of JMJD8 protein level but also led to a significant reduction of TNF-induced expression of *IL8* and *TNFα* transcripts (Fig. 1b, upper panel). The same effect was observed with JMJD8 knockdown in HONE1 (Nasopharyngeal carcinoma cells), HaCat (Immortalized human keratinocytes) and U2OS (Osteosarcoma cells) cell lines indicating that the observed defects in NF-κB activation caused by JMJD8 knockdown is not cell-type specific (see Supplementary Fig. S2). In addition, we attempted to rescue the defective TNF-induced NF-κB activation by reconstituting the JMJD8 knockdown HEK293T cells with a siRNA-resistant JMJD8 transcript. We demonstrated that transient over-expression of the siRNA-resistant JMJD8 in JMJD8 knockdown HEK293T cells leads to significant recovery of both *IL8* and *TNFα* expression in a dose-dependent manner albeit less pronounced in *IL8* suggesting that JMJD8 is indeed a positive regulator of TNF-induced NF-κB signaling (Fig. 1c). Surprisingly, we did not see any enhanced NF-κB activation when JMJD8 was transiently over-expressed in 293T-luc cells, with or without TNFα stimulation (see Supplementary Fig. S1b).

To determine whether the observed defect in NF-κB activation caused by JMJD8 knockdown is specific to TNF-induced NF-κB signaling, we infected the control and JMJD8 knockdown HEK293T cells with and without Sendai virus. Surprisingly, JMJD8 knockdown markedly suppressed IFNβ induction by Sendai virus infection (Fig. 1d). This observation suggests that JMJD8 may be involved in other pathways and may not be specifically restricted to TNF-induced NF-κB signaling.

### JMJD8 deficiency reduces TNF-induced IκBα degradation and p65 translocation

**Figure 2:**
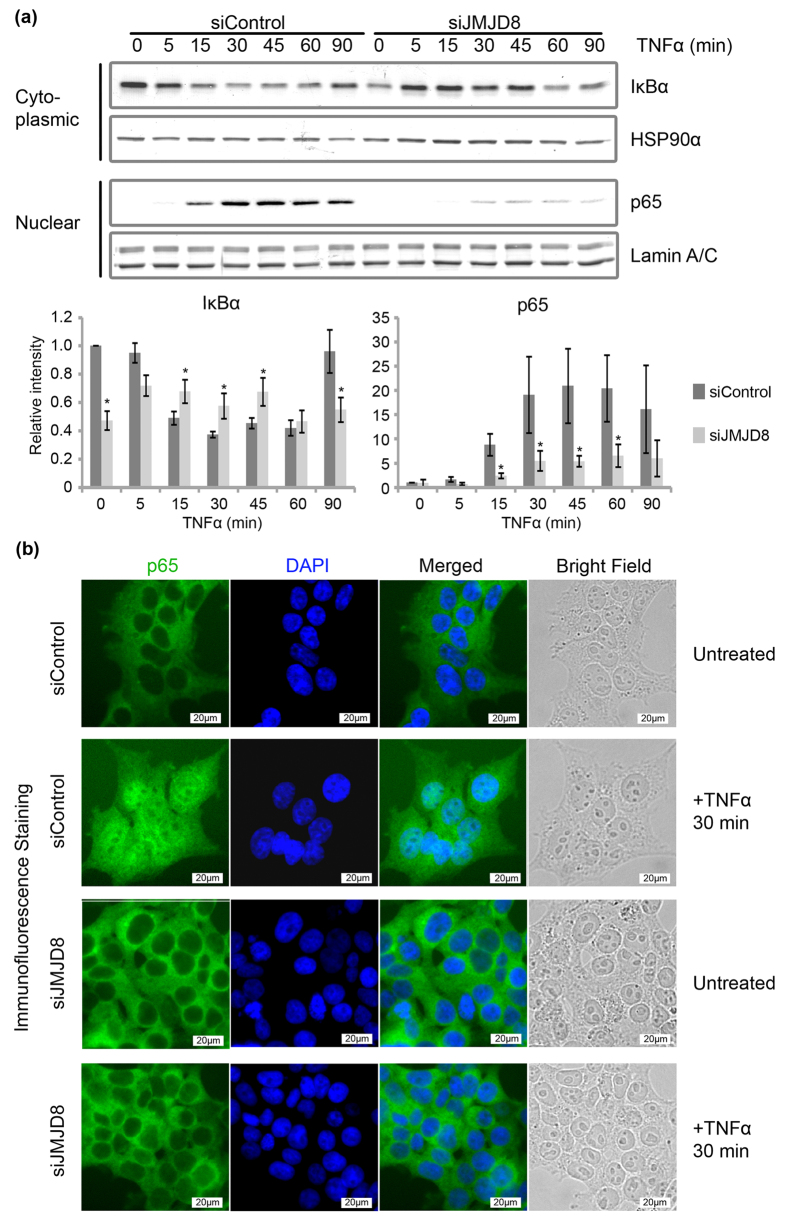
JMJD8 deficiency reduces TNF-induced IκBα degradation and p65 translocation. (**a**) Control and JMJD8 knockdown HEK293T cells were treated with 10 ng/ml of TNFα for 0, 5, 15, 30, 45, 60 and 90 minutes. Cytoplasmic and nuclear fractions were prepared and immunoblotted for IκBα and p65. HSP90α and Lamin A/C were used as cytoplasmic and nuclear loading controls respectively. Relative intensity of bands were quantified using the Image Lab (BioRad)/ImageJ software, were normalized to HSP90α or Lamin A/C, and shown in relative to 0 minute of siControl (n = 3). (**b**) Control and JMJD8 knockdown HEK293T cells were treated with 10 ng/ml of TNFα for 30 minutes. P65 localization was visualized with an immunofluorescence assay. Images were acquired with an Olympus IX71 fluorescence microscope. Scale bar: 20 μm. (n = 3). Data represent means ± SD. (*p > 0.05). Full-length blots are presented in Supplementary Fig. S4.

To dissect the role of JMJD8 in the TNF pathway, we first investigated the degradation of IκBα and the nuclear translocation of NF-κB, which are the two biochemical hallmarks of NF-κB activation. HEK293T cells were transfected with control or JMJD8-targeting siRNA oligos and treated with TNFα at the indicated time points. The cells were harvested and fractionated into cytoplasmic and nuclear fractions. TNF-induced degradation of IκBα peaked at 30 minutes followed by a resynthesis of IκBα at 90 minutes in the control cells (Fig. 2a upper panel). We found that IκBα degradation was reduced or delayed in the JMJD8-deficient cells and no resynthesis of IκBα was observed. Consistent with impaired IκBα degradation, we observed a significant reduction of TNF-induced p65 nuclear translocation in JMJD8-deficient cells (Fig. 2a, lower panel). To further confirm this observation, we performed an immunofluorescence assay to visualize the p65 subcellular localization in control and JMJD8 knockdown HEK293T cells with and without TNFα stimulation. Consistently, we noticed a complete blockage of p65 translocation into the nuclear of JMJD8 knockdown cells (Fig. 2b), indicating that JMJD8 is required for both IκBα degradation and the release of NF-κB into nucleus.

### JMJD8 is essential for IKK kinase activation

**Figure 3:**
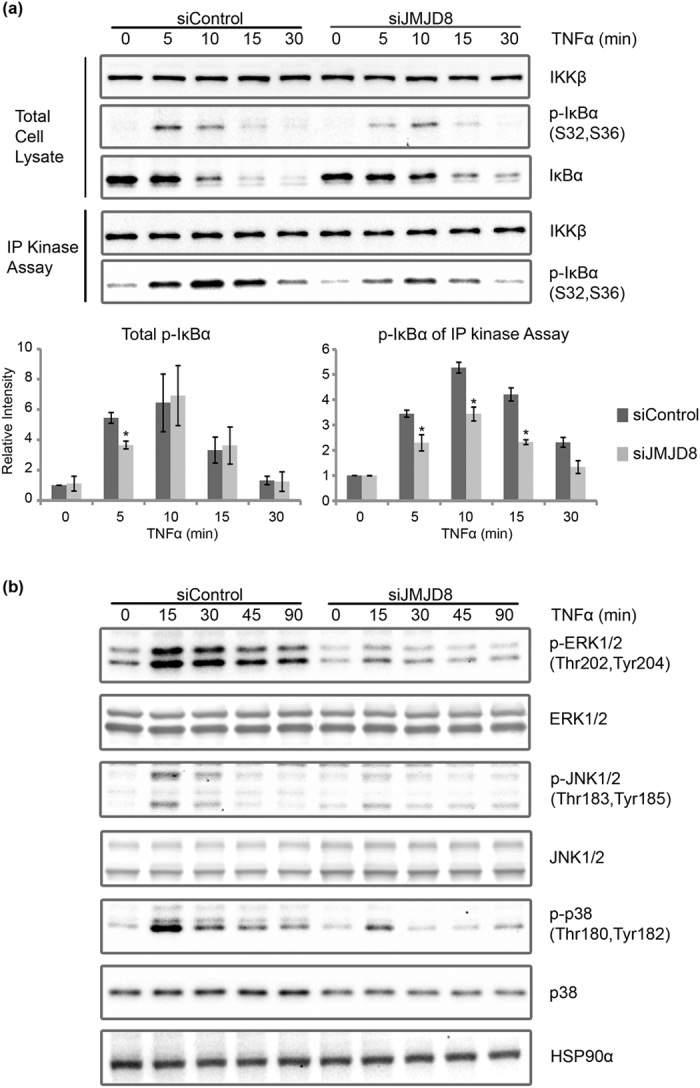
JMJD8 is required for TNF-induced IKK kinase activity. (**a**) Control and JMJD8 knockdown HEK293T cells were induced with 10 ng/ml of TNFα for 0, 5, 10, 15 and 30 minutes. IKK kinase activity was measured with an *in vitro* kinase assay followed by immunoblotting using the anti-p-IκBα and anti-IKKβ antibodies. Relative intensity of bands were quantified using the Image Lab (BioRad)/ImageJ, were normalized to IKKβ, and shown in relative to 0 minute of siControl (n = 2). (**b**) Control and JMJD8 knockdown HEK293T cells were induced with 10 ng/ml of TNFα for 0, 15, 30, 45 and 90 minutes. Total cell lysates were prepared and immunoblotted with the indicated antibodies (n = 2). Data represent means ± SD. (*p > 0.05). Full-length blots are presented in Supplementary Figs S5–S8, respectively.

The observed defect in IκBα degradation in JMJD8 knockdown cells suggests that JMJD8 may regulate the upstream signal transduction of TNF pathway. IκBα phosphorylation by IKK complexes is a prerequisite step for IκBα degradation^7^. Therefore, we investigated the activation of IKK in the presence or absence of JMJD8. We treated the control and JMJD8 knockdown HEK293T cells with TNFα at indicated time points and measured the IKK kinase activity with an *in vitro* IKK kinase assay. IKK kinase activity was detected as early as 5 minutes post-TNF stimulation and peaked at 10 minutes (Fig. 3a, lower panel). In the absence of JMJD8, TNF-induced IKK activation was significantly reduced as measured by the *in vitro* IKK kinase assay as well as the immunoblotting of p-IκBα in the total cell extracts (Fig. 3a, upper panel). This result suggests that JMJD8 is required for TNF-induced activation of IKK.

In parallel to the activation of NF-κB, TNF also activates the MAPK pathways, including ERK, JNK and p38 pathways^22^. To examine whether TNF-induced MAPK pathways are affected in JMJD8 knockdown cells, we checked the activation status of MAPKs in response to TNF stimulation. Unexpectedly, activation of MAP kinases was also reduced in JMJD8 knockdown cells (Fig. 3b). There was a significant reduction of the phosphorylation of JNK1/2 (p-JNK1/2), ERK1/2 (p-ERK1/2) and p38 (p-p38) in JMJD8 knockdown cells compared to the control cells. These results suggest that JMJD8 is required for the activation of MAPKs in response to TNF stimulation.

### JMJD8 is required for IKK activation and RIP1 ubiquitination

**Figure 4:**
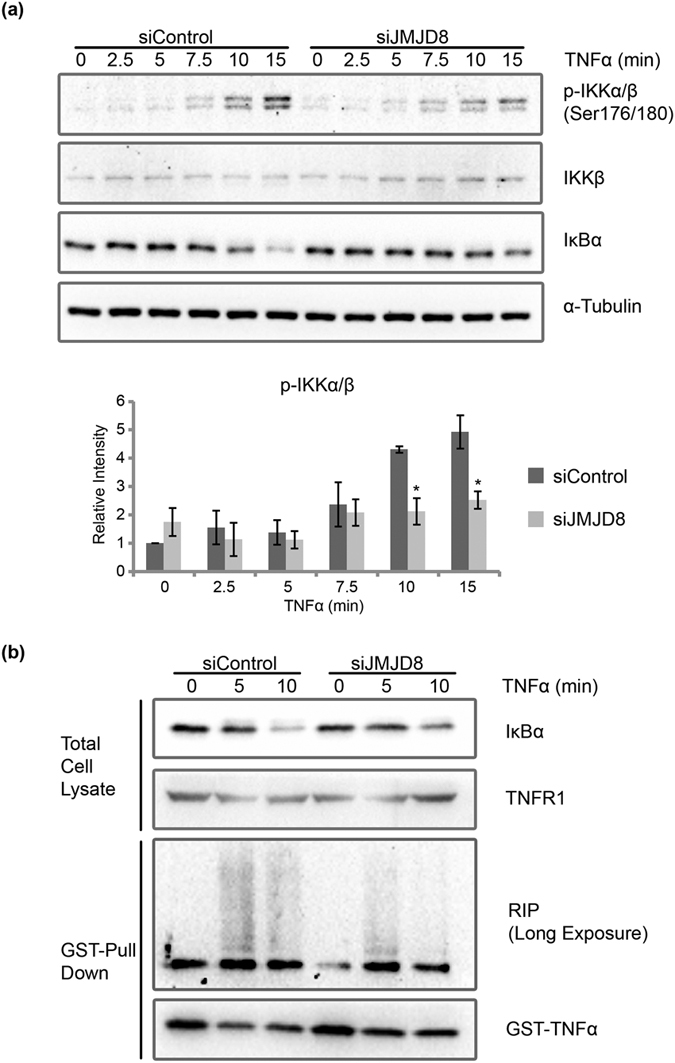
JMJD8 is required for IKK activation and RIP1 ubiquitination. (**a**) Control and JMJD8 knockdown HEK293T cells were induced with 10 ng/ml of TNFα for 0, 2.5, 5, 7.5, 10 and 15 minutes. Total cell lysates were prepared and immunoblotted with the indicated antibodies. Relative intensity of bands were quantified using the Image Lab (BioRad)/ImageJ, were normalized to IKK or α-Tubulin, and shown in relative to 0 minute of siControl (n = 2). (**b**) Control and JMJD8 knockdown HEK293T cells were induced with 1 μg/ml of GST-TNFα for 0, 5 and 10 minutes. TNFR1 complexes were pulled down with Glutathione beads and immunoblot for RIP1 and TNFα (n = 10). Data represent means ± SD. (*p > 0.05). Full-length blots are presented in Supplementary Figs S9 and S10, respectively.

Phosphorylation of IKK at Serine 177 and Serine 181 in the activation loop of IKKβ (Serine 176 and Serine 180 in IKKα) is required for its kinase activity^23^. To investigate the phosphorylation status of IKK, we therefore treated control and JMJD8-deficient cells with TNFα at indicated time points and measured the amount of p-IKK. Consistent with the IKK kinase assay, p-IKK was significantly lesser in JMJD8 knockdown cells (Fig. 4a).

RIP1 ubiquitination is another key event that is essential for TNF-induced NF-κB signaling^24^,^25^. To examine whether RIP1 ubiquitination is affected in JMJD8-deficient cells, we pulled down the TNFR1 receptor complex from control and JMJD8 knockdown HEK293T cells that was treated with and without GST-TNFα and examined the RIP1 ubiquitination by immunoblotting with specific antibody against RIP1. Interestingly, we noticed a significant reduction of RIP1 ubiquitination in JMJD8 knockdown cells (Fig. 4b) suggesting that JMJD8 may regulate the upstream components of TNF-induced NF-κB signaling. However, no interaction between RIP1 and JMJD8 was detected with a co-immunoprecipitation assay (see Supplementary Fig. S12).

### JMJD8 deficiency favors cells towards TNF-induced apoptosis

**Figure 5:**
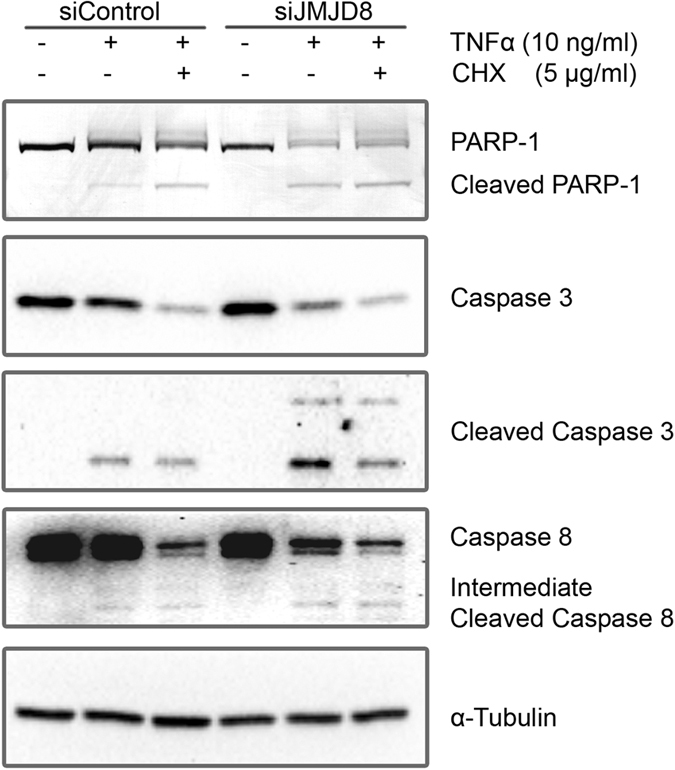
JMJD8 deficiency promotes TNF-induced apoptosis. (**a**) Control and JMJD8 knockdown HEK293T cells were treated with either 10 ng/ml of TNFα alone or together with 5 μg/ml of Cyclohexamide (CHX) for 12 hours. Total cell lysates were prepared and immunoblotted with the indicated antibodies (n = 3). Full-length blots are presented in Supplementary Fig. S11.

TNFα is a pleiotropic cytokine which can lead to two distinct cell fates which are the pro-survival path, mainly through the activation of pro-survival genes by NF-κB, or pro-apoptotic path through the signaling cascade of caspases activation^4^. We hypothesized that the defects in pro-survival path will favor the cells towards pro-apoptotic pathway. To investigate this speculation, we treated control and JMJD8 knockdown HEK293T cells with and without TNFα and examined apoptosis by immunoblotting total cell lysates with specific antibodies against caspase 3, cleaved-caspase 3, caspase 8 and PARP. TNF-only treatment induced a moderate level of apoptosis in control cells. Apoptosis was further enhanced in the presence of both TNF and cyclohexamide (CHX) as evidenced by the presence of cleaved PARP, reduced level of pro-caspase 3 and 8, and increased level of cleaved-caspase 3 and intermediate cleaved-caspase 8 (Fig. 5). In the absence of JMJD8, TNF-only treatment induced high level of apoptosis that was comparable to the control cells treated with both TNF and CHX. Collectively, these results indicate that JMJD8 is required for the pro-survival pathway of TNF-induced NF-κB signaling.

## Discussion

To the best of our knowledge, this is the first report that demonstrates a functional role of JMJD8 in TNF-induced NF-κB signaling. We show that knockdown of JMJD8 expression in HEK293T cells results in reduced TNF-induced NF-κB-dependent genes expressions, IκBα degradation and p65 nuclear translocation. The upstream RIP1 ubiquitination, phosphorylation of IKK, IKK kinase activity, and MAP kinase activation are also suppressed with the depletion of JMJD8 expression. Furthermore, TNF-induced apoptosis is enhanced in the absence of JMJD8.

IκBα degradation is regulated by the phosphorylation of its two serine residues by IKK kinase^26^,^27^,^28^. Our results suggest that lesser IKK kinases are activated in JMJD8-deficient cells. Furthermore, in parallel with our *in vitro* kinase assay, we observed lesser phosphorylation of IKK in JMJD8-silenced cells (Fig. 4a). Consequently, lesser phosphorylation of IκBα leads to lesser IκBα degradation (Fig. 3a). In addition, TNF-induced MAPK pathways are defective in JMJD8-deficient cells (Fig. 3b). Moreover, TNF induced less ubiquitination of RIP1 in the absence of JMJD8. Co-immunoprecipitation assay suggests that RIP1 does not interact with JMJD8. Thus, we suspect that the defect in NF-κB activation in JMJD8 knockdown cells lies upstream or at the level of RIP1 ubiquitination. Interestingly, depletion of JMJD8 also greatly suppressed Sendai virus-induced *IFNβ* expression which suggests that JMJD8 may be essential for the type I interferon pathway as well. On the contrary, overexpression of JMJD8 however did not lead to enhanced NF-κB activation in 293T-luc cells.

Several studies have previously reported the involvement of different methylation of p65 subunit in NF-κB signaling pathway^9^,^10^,^11^,^29^,^30^,^31^,^32^,^33^,^34^. However, our reduced IKK kinase activity and p-IKK results (Figs 3a and 4a, respectively) in JMJD8 knockdown cells would argue against the possibility of p65 subunit, which is downstream of IKK, being the regulatory target of JMJD8. On the other hand, TRAF proteins for example TRAF2, 5 and 6 are adaptors for activation of various NF-κB signaling pathways. These proteins have been implicated to act as essential intermediates during TNFR complex formation in TNF-induced signaling as well as during virus infection-dependent NF-κB activation^35^,^36^. Furthermore, JMJD6 which is structurally highly similar to JMJD8, was reported to demethylate TRAF6 in response to Toll-like receptor ligands^37^. Therefore, it is possible that JMJD8 may regulate TRAF proteins similar to JMJD6 in TNF-induced signaling and antiviral response in the cytoplasm. Recent study by Boeckel *et al*. have shown that JMJD8 localized to the extranuclear region rather than the nuclear compartment and interact with phosphofructokinase 1, JAK1, CANX and PKM2 cytoplasmic proteins thus, suggesting that the substrate of JMJD8 may be different and unconventional^38^. These observations further strengthen our results that JMJD8 may regulate protein outside of nuclear which is different from typical Jumonji domain-containing proteins that target the histone. However, it remains to be determined whether JMJD8 possesses any demethylase enzymatic activity which should be an exciting subject for future study. There is some evidence beginning to show that Jumonji domain-containing proteins function without its demethylase enzymatic activity in the cells. For example, JMJD3 was found to act as an adaptor for PHF20 to recruit Trim26, an E3 ligase for K48-linked polyubiquitination and mediates PHF20 proteasomal degradation^39^.

Our results show that JMJD8 depletion promotes TNF-induced apoptosis (Fig. 5). In general, TNFR1 is able to form two distinct complexes that lead to different cell fates. While TNFR1 complex I is important for pro-survival, TNFR1 complex II is required for pro-apoptotic function^5^. Based on our results, the shift in the activation of TNFR1 complex I to II that eventually leads to enhanced apoptosis in JMJD8-deficient cells may be due to the failure or inability of TNFR1 complex I to form appropriately. This is further supported by the reduction of RIP1 ubiquitination in JMJD8-silenced cells (Fig. 4b) which is consistent with previous studies that RIP1 ubiquitination is essential for TNF-induced NF-κB signaling^40^,^41^,^42^. In contrast, non-ubiquitinated RIP1 would serve as pro-apoptotic signaling molecule that recruits caspase 8 to the TNFR1 complex^43^. As a result, the incomplete activation of pro-survival pathway may lead to the promotion of pro-apoptotic pathway. Together, these results imply that JMJD8 may be required for TNFR1 complex I formation.

In conclusion, we show that JMJD8 acts as a positive regulator in TNF-induced NF-κB signaling. However, the precise mechanism of action and target of JMJD8 remain unknown. Further studies will be required to pin point the exact target of JMJD8 to fully elucidate its role in TNF-induced NF-κB signaling.

## Methods

### Cell culture

HEK293T, HONE1, HaCat and U2OS cells were maintained in Dulbecco’s modified Eagle’s medium (DMEM; Gibco) supplemented with 10% (v/v) FBS, 100 IU/ml penicillin and 100 μg/ml streptomycin (Gibco).

### Reagents and antibodies

Recombinant TNFα was purchased from Gold Biotechnology (St. Louis, MO). Antibodies against TNFR1 (H-271), NEMO (FL-419), IKKβ (C-20), IκBα (C-21), p65 (C-20 and F-6), HSP90α (C-20), α-Tubulin (TU-02), PARP-1 (F-2), Caspase 8 (C-20), Caspase 3 (E-8), JNK (D-6 and N-18), ERK1 (K-23), ERK2 (C-14), p38 (H-174) and c-Myc (9E10) were acquired from Santa Cruz Biotechnology; whereas, cleaved-caspase 3 (5A1E), RIP1 (D94C12) and phosphorylated form of JNK, ERK, p38 (D3F9), IKKα/β (16A6), FLAG (9A3) and IκBα were bought from Cell Signaling Technology. Anti-JMJD8 was purchased from Abnova.

### Mammalian and bacterial expression vectors

Human transcript of JMJD8 was cloned into pcDNA3 vector (Invitrogen) to generate pcDNA3-hJMJD8. To remove the siRNA targeting site, a site directed mutagenesis was conducted to generate silence mutations at 3 nucleotides (pcDNA3-hJMJD8-siJMJD8*). The plasmids were subsequently verified by automated DNA sequencing. GST-IκBα was expressed in Top10 cells and purified with Glutathione beads according to the manufacturer’s recommendation.

### siRNA

The siRNAs for hJMJD8 were purchased from Sigma (SASI_Hs02_00305057 and SASI_Hs01_00228274). The control siRNAs (D-001810-10-50) were purchased from Dharmacon. The siRNAs were transfected into HEK293T cells via calcium phosphate precipitation at a final concentration of 20 nM on the first and second day to enhance knockdown efficiency. Media was changed on the third day and incubated overnight before treating the cells with and without TNFα for the indicated time points.

### RNA isolation and qPCR

Total RNA was prepared with the Thermo Scientific GeneJET RNA Purification Kit according to the manufacturer’s protocol. The cDNA was synthesized from 0.5–1 μg of RNA (DNase I-treated, Thermoscientific) using random hexamer (Invitrogen), dNTPs (Thermoscientific), RNase inhibitor and Moloney Murine Leukemia Virus (M-MuLV, MMLV) Reverse Transcriptase (NEB) according to the manufacturer’s recommendation. Generated cDNA was used for subsequent RT-qPCR assays. The RT-qPCR was carried out with indicated primers and KAPA SYBR FAST qPCR Master Mix (Kapa Biosystem) according to the manufacturer’s protocol. All data were then normalized to SDHA. The primer sequences are listed in Supplementary Table S1.

### Subcellular fractionation

To examine the nuclear translocation of NF-κB, the cells were fractionated into cytoplasmic and nuclear fractions. Briefly, HEK293T cells treated with or without TNFα were washed 3 times with 1× PBS and lysed with a hypotonic lysis buffer (10 mM Tris, pH 7.5; 1.5 mM MgCl_2_; 10 mM KCl; 0.5 mM DTT; 0.5 mM PMSF; 1 × Protease Inhibitor, 0.05% NP40). The nuclear was isolated by centrifugation at 500 g, 4 °C for 5 minutes and resuspended in a nuclear lysis buffer (25 mM Tris, pH 7.5; 420 mM NaCl; 1.5 mM MgCl_2_; 0.2 mM EDTA; 25% Glycerol; 0.5 mM DTT; 0.5 mM PMSF; 1 × Protease Inhibitor). Debris from both cytoplasmic and nuclear fractions was cleared by centrifugation at max speed at 4 °C for 5 minutes.

### IKK kinase Assay

To study the IKK kinase activity, the IKK complex was immunoprecipitated from control and JMJD8 knockdown HEK293T cells which were treated with TNFα at the indicated time points. Briefly, the cells were lysed in an IPKA lysis buffer (20 mM Tris pH 7.5, 150 mM NaCl, 10% glycerol, 25 mM β-glycerol-phosphate, 1 mM orthovanadate, 1 mM DTT, 1 mM PMSF, 1% triton X100) and total protein level was quantitated using Bradford assay. Next, 500 μg of total proteins were immunoprecipitated with an anti-IKKγ (sc-8330) antibody and 15 μl of 50% slurry protein A/G beads for 1 hour at 4 °C, then washed with IPKA lysis buffer twice and kinase assay buffer without ATP for the third wash. The beads were incubated with 200 ng of GST-IκBα in 1 × kinase buffer (20 mM Hepes pH 7.5, 50 mM NaCl, 20 mM β-glycerol-phosphate, 10 mM MgCl_2_, 1 mM orthovanadate, 200 μM ATP) for 30 minutes at 30 °C. After the incubation, the products were analysed with immunoblotting using an anti- p-IκBα antibody.

### TNFR1 recruitment assay

The control and JMJD8-knockdown cells were induced with 1 μg/ml of GST-TNFα for the indicated time points, washed with ice-cold 1× PBS for three times and lysed in IPKA lysis buffer. Next, the lysates were preclear with protein A/G beads on a rotator for 1 hour at 4 °C. TNFR1 complexes were pulled down with Glutathione beads and bound proteins were analyzed with immunoblotting using the indicated antibodies.

### Immunofluorescence assay

To examine the protein localization, cells were fixed with 4% formaldehyde for 15 minutes and then permeabilized and blocked with 1× PBS supplemented with 5% fetal bovine serum and 0.3% Triton X-100 for 30 minutes. Then, cells were incubated overnight with the primary antibodies according to manufacturer’s recommended dilution. Next, cells were washed 3 times with 1× PBS followed by 1 hour incubation with specific AlexaFluor-conjugated secondary antibodies (Cell Signaling). Images were acquired with a Olympus IX71 fluorescent microscope with a 40x objective. Images were analysed using the cell sens standard and FV10-ASW viewer softwares (Olympus).

### Immunoprecipitation

To examine the interaction between RIP1 and JMJD8, HEK293T cells were transfected with RIP1 and JMJD8-expressing constructs. The cells were lysed in the IPKA lysis buffer and quantified as previously described. Five hundred microgram of total proteins were immunoprecipitated with an anti-c-Myc (sc-40), FLAG (8146 S) or mouse IgG antibody, and 10 μl of 50% slurry protein A/G beads (Pierce) for overnight at 4 °C, then washed with the IPKA lysis buffer for 4 times. Bound proteins were analyzed with immunoblotting using the indicated antibodies. Thirty microgram of TCL was included as positive control.

### Luciferase assay

Stable HEK293T cells carrying a luciferase reporter driven by NF-κB enhancer found in immunoglobulin kappa light chain gene (293T-luc cells) were transfected with siRNA for knockdown study or JMJD8-expressing vector for overexpression study before treating the cells with and without TNFα (10 ng/ml) for an additional 12 hours. Cells were lysed in luciferase lysis buffer (100 mM Sodium Phosphate buffer pH7.8, 8 mM MgCl_2_, 1 mM DTT, 1% Triton X-100 and 15% glycerol) and the luciferase activities were measured using a TECAN M200 plate reader according to the manufacturer’s instructions.

### Statistical analysis

Data were analyzed with Microsoft Excel and presented as mean ± SD. Data are representative of two or more independent experiments. Statistical significance was assessed using two-tailed unpaired Student’s t-test.

## Additional Information

**How to cite this article**: Yeo, K. S. *et al*. JMJD8 is a positive regulator of TNF-induced NF-κB signaling. *Sci. Rep.* **6**, 34125; doi: 10.1038/srep34125 (2016).

## Supplementary Material

Supplementary Information

## References

[b1] Aggarwal B. B., Gupta S. C. & Kim J. H. Historical perspectives on tumor necrosis factor and its superfamily: 25 years later, a golden journey. Blood 119, 651–665 (2012). 22053109 10.1182/blood-2011-04-325225 PMC3265196

[b2] Hehlgans T. & Pfeffer K. The intriguing biology of the tumour necrosis factor/tumour necrosis factor receptor superfamily: Players, rules and the games. Immunology 115, 1–20 (2005). 15819693 10.1111/j.1365-2567.2005.02143.x PMC1782125

[b3] Wertz I. E. TNFR1-activated NF-κB signal transduction: regulation by the ubiquitin/proteasome system. Current opinion in chemical biology 23, 71–7 (2014). 25461388 10.1016/j.cbpa.2014.10.011

[b4] Brenner D., Blaser H. & Mak T. W. Regulation of tumour necrosis factor signalling: live or let die. Nat. Rev. Immunol. 15, 362–374 (2015). 26008591 10.1038/nri3834

[b5] Micheau O. & Rg Tschopp J. Induction of TNF Receptor I-Mediated Apoptosis via Two Sequential Signaling Complexes. Cell 114, 181–190 (2003). 12887920 10.1016/s0092-8674(03)00521-x

[b6] Hayden M. S. & Ghosh S. Shared principles in NF-kappaB signaling. Cell 132, 344–362 (2008). 18267068 10.1016/j.cell.2008.01.020

[b7] Hayden M. S. & Ghosh S. Regulation of NF-κB by TNF family cytokines. Seminars in immunology 26, 253–66 (2014). 24958609 10.1016/j.smim.2014.05.004 PMC4156877

[b8] Silverman N. & Maniatis T. NF-kappaB signaling pathways in mammalian and insect innate immunity. Genes Dev. 15, 2321–2342 (2001). 11562344 10.1101/gad.909001

[b9] Carr S. M., Roworth A. P., Chan C. & Thangue N. B. La. Post-translational control of transcription factors: methylation ranks highly. FEBS J. 282, 4450–4465 (2015). 26402372 10.1111/febs.13524

[b10] Ea C.-K. & Baltimore D. Regulation of NF-kappaB activity through lysine monomethylation of p65. Proc. Natl. Acad. Sci. USA 106, 18972–18977 (2009). 19864627 10.1073/pnas.0910439106 PMC2770010

[b11] Perkins N. D. Post-translational modifications regulating the activity and function of the nuclear factor kappa B pathway. Oncogene 25, 6717–6730 (2006). 17072324 10.1038/sj.onc.1209937

[b12] Chen L. F., Mu Y. J. & Greene W. C. Acetylation of RelA at discrete sites regulates distinct nuclear functions of NF—KB. EMBO J 21, 6539–6548 (2002). 12456660 10.1093/emboj/cdf660 PMC136963

[b13] Kiernan R. . Post-activation turn-off of NF-kappa B-dependent transcription is regulated by acetylation of p65. J. Biol. Chem. 278, 2758–2766 (2003). 12419806 10.1074/jbc.M209572200

[b14] Lu T. . Regulation of NF-κB by NSD1/FBXL11-dependent reversible lysine methylation of p65. Proc. Natl. Acad. Sci. USA 107, 46–51 (2010). 20080798 10.1073/pnas.0912493107 PMC2806709

[b15] Lu T. . Validation-based insertional mutagenesis identifies lysine demethylase FBXL11 as a negative regulator of NFkappaB. Proc. Natl. Acad. Sci. USA 106, 16339–16344 (2009). 19805303 10.1073/pnas.0908560106 PMC2736141

[b16] Takeuchi T. . Gene trap capture of a novel mouse gene, jumonji, required for neural tube formation. Genes Dev. 9, 1211–1222 (1995). 7758946 10.1101/gad.9.10.1211

[b17] Yamane K. . JHDM2A, a JmjC-containing H3K9 demethylase, facilitates transcription activation by androgen receptor. Cell 125, 483–95 (2006). 16603237 10.1016/j.cell.2006.03.027

[b18] Tsukada Y. . Histone demethylation by a family of JmjC domain-containing proteins. Nature 439, 811–816 (2006). 16362057 10.1038/nature04433

[b19] Shi Y. & Whetstine J. R. Dynamic regulation of histone lysine methylation by demethylases. Mol. Cell 25, 1–14 (2007). 17218267 10.1016/j.molcel.2006.12.010

[b20] Yang Z. Q. . Identification of a novel gene, GASC1, within an amplicon at 9p23-24 frequently detected in esophageal cancer cell lines. Cancer Res. 60, 4735–9 (2000). 10987278

[b21] Cloos P. a. C. . The putative oncogene GASC1 demethylates tri- and dimethylated lysine 9 on histone H3. Nature 442, 307–11 (2006). 16732293 10.1038/nature04837

[b22] Karin M. & Gallagher E. TNFR signaling: ubiquitin-conjugated TRAFfic signals control stop-and-go for MAPK signaling complexes. Immunol. Rev. 228, 225–240 (2009). 19290931 10.1111/j.1600-065X.2008.00755.x

[b23] Delhase M., Hayakawa M., Chen Y. & Karin M. Positive and negative regulation of IkappaB kinase activity through IKKbeta subunit phosphorylation. 284, 309–313 (1999). 10.1126/science.284.5412.309 10195894

[b24] Li H., Kobayashi M., Blonska M., You Y. & Lin X. Ubiquitination of RIP Is Required for Tumor Necrosis Factor α-induced NF-κB Activation. J. Biol. Chem. 281, 13636–13643 (2006). 16543241 10.1074/jbc.M600620200

[b25] Ea C. K., Deng L., Xia Z. P., Pineda G. & Chen Z. J. Activation of IKK by TNFα Requires Site-Specific Ubiquitination of RIP1 and Polyubiquitin Binding by NEMO. Molecular Cell 22, 245–257 (2006). 16603398 10.1016/j.molcel.2006.03.026

[b26] Brown K., Gerstberger S., Carlson L., Franzoso G. & Siebenlist U. Control of I kappa B-alpha proteolysis by site-specific, signal-induced phosphorylation. Science (80-.). 267, 1485–1488 (1995). 10.1126/science.7878466 7878466

[b27] Chen Z. . Phosphorylation targets IKB x to the ubiquitin-proteasome pathway. Genes Dev. 9, 1586–1598 (1995). 7628694 10.1101/gad.9.13.1586

[b28] Scherer D. C., Brockman J. A., Chen Z., Maniatis T. & Ballard D. W. Signal-induced degradation of I kappa B alpha requires site-specific ubiquitination. Proc. Natl. Acad. Sci. USA 92, 11259–63 (1995). 7479976 10.1073/pnas.92.24.11259 PMC40611

[b29] Levy D., Kuo A., Chang Y. & Schaefer U. SETD6 lysine methylation of RelA couples GLP activity at chromatin to tonic repression of NF-κB signaling. Nat. Immunol. 12, 29–36 (2011). 21131967 10.1038/ni.1968 PMC3074206

[b30] Harris D. P., Bandyopadhyay S., Maxwell T. J., Willard B. & DiCorleto P. E. TNF-α induction of CXCL10 in endothelial cells requires protein arginine methyltransferase 5 (PRMT5)-mediated NF-κB p65 Methylation. J. Biol. Chem. 289, 15328–15339 (2014). 24753255 10.1074/jbc.M114.547349 PMC4140890

[b31] Chang Y. . Structural basis of SETD6-mediated regulation of the NF-kB network via methyl-lysine signaling. Nucleic Acids Res. 39, 6380–6389 (2011). 21515635 10.1093/nar/gkr256 PMC3159447

[b32] Lu T. . Role of lysine methylation of NF-κB in differential gene regulation. Proceedings of the National Academy of Sciences of the United States of America 110, 13510–5 (2013). 23904479 10.1073/pnas.1311770110 PMC3746872

[b33] Yang X.-D., Tajkhorshid E. & Chen L.-F. Functional interplay between acetylation and methylation of the RelA subunit of NF-κB. Mol. Cell. Biol. 30, 2170–2180 (2010). 20160011 10.1128/MCB.01343-09 PMC2863596

[b34] Yang X.-D. . Negative regulation of NF-κB action by Set9-mediated lysine methylation of the RelA subunit. EMBO J. 28, 1055–66 (2009). 19262565 10.1038/emboj.2009.55 PMC2683704

[b35] Gil J. . TRAF Family Proteins Link PKR with NF-κB Activation. Mol. Cell. Biol. 24, 4502–4512 (2004). 15121867 10.1128/MCB.24.10.4502-4512.2004 PMC400457

[b36] Liu S. . MAVS recruits multiple ubiquitin E3 ligases to activate antiviral signaling cascades. Elife 2, e00785 (2013). 23951545 10.7554/eLife.00785 PMC3743401

[b37] Tikhanovich I. . Dynamic arginine methylation of TNF receptor associated factor 6 regulates Toll-like receptor signaling. J. Biol. Chem. 290, 22236–22249 (2015). 26221041 10.1074/jbc.M115.653543 PMC4571974

[b38] Boeckel J.-N. . JMJD8 Regulates Angiogenic Sprouting and Cellular Metabolism by Interacting With Pyruvate Kinase M2 in Endothelial Cells. Arterioscler. Thromb. Vasc. Biol. ATVBAHA. 116.307695, 10.1161/ATVBAHA.116.307695 (2016). 27199445

[b39] Zhao W. . Jmjd3 Inhibits Reprogramming by Upregulating Expression of INK4a/Arf and Targeting PHF20 for Ubiquitination. Cell 152, 1037–1050 (2013). 23452852 10.1016/j.cell.2013.02.006 PMC3742052

[b40] Legler D. F., Micheau O., Doucey M. A., Tschopp J. & Bron C. Recruitment of TNF Receptor 1 to Lipid Rafts Is Essential for TNFα-Mediated NF-κB Activation. Immunity 18, 655–664 (2003). 12753742 10.1016/s1074-7613(03)00092-x

[b41] Kanayama A. . TAB2 and TAB3 activate the NF-κB pathway through binding to polyubiquitin chains. Molecular Cell 15, 535–548 (2004). 15327770 10.1016/j.molcel.2004.08.008

[b42] Lee T. H., Shank J., Cusson N. & Kelliher M. a. The kinase activity of Rip1 is not required for tumor necrosis factor-alpha-induced IkappaB kinase or p38 MAP kinase activation or for the ubiquitination of Rip1 by Traf2. The Journal of biological chemistry 279, 33185–33191 (2004). 15175328 10.1074/jbc.M404206200

[b43] O’Donnell M. A., Legarda-Addison D., Skountzos P., Yeh W. C. & Ting A. T. Ubiquitination of RIP1 Regulates an NF-κB-Independent Cell-Death Switch in TNF Signaling. Curr. Biol. 17, 418–424 (2007). 17306544 10.1016/j.cub.2007.01.027 PMC1868513

