# Supplementary material for: JMJD8 is a positive regulator of TNF-induced NF-κB signaling
Source: Sci Rep. 2016 Sep 27;6:34125. doi: 10.1038/srep34125 (PMC5037431; doi:10.1038/srep34125)
Supplement: Supplementary Information [file srep34125-s1.pdf]

Supplementary Materials

JMJD8 is a positive regulator of TNF-induced NF-κB signaling.

Kok Siong Yeo [1], Ming Cheang Tan [1], Wan Ying Wong [1], Sheng Wei Loh [1], Yi Lyn Lam [1] ,

Chin Leng Tan [1], Yat-Yuen Lim [1,]*, Chee-Kwee Ea [1,2]*

Author information

Affiliations

1 Institute of Biological Sciences, Faculty of Science,

University of Malaya,

50603 Kuala Lumpur, Malaysia

2 Current address: Department of Molecular Biology,

University of Texas Southwestern Medical Center,

Dallas, TX 75390-9148, United States

*To whom correspondence should be addressed:

yatyuen.lim@um.edu.my; Chee-Kwee.Ea@utsouthwestern.edu

Phone: 603-79677022ext2587, Fax: 603-79557727

Supplementary Table 1: List of primers used in qPCR assays.

Genes	Primers Sequence 5' – 3'
IL8 Forward	AGCTCTGTCTGGACCCCAAG
IL8 Reverse	GAATTCTCAGCCCTCTTCAAAAAC
TNFα Forward	GCCCAGGCAGTCAGATCATCT
TNFα Reverse	TTGAGGGTTTGCTACAACATGG
IκBα Forward	CTCCGAGACTTTCGAGGAAATAC
IκBα Reverse	GCCATTGTAGTTGGTAGCCTTCA
CCL5 Forward	CCCAGCAGTCGTCTTTGTCA
CCL5 Reverse	TCCCGAACCCATTTCTTCTCT
IFNβ Forward	ACTGCCTCAAGGACAGGATG
IFNβ Reverse	AGCCAGGAGGTTCTCAACAA
JMJD8 Forward	GACAGGTTGCTGGCTTCGTT
JMJD8 Reverse	AGGGCAAGTCCACTTTGTGGTA
SDHA Forward	TGGGAACAAGAGGGCATCTG
SDHA Reverse	CCACCACTGCATCAAATTCATG

(a)

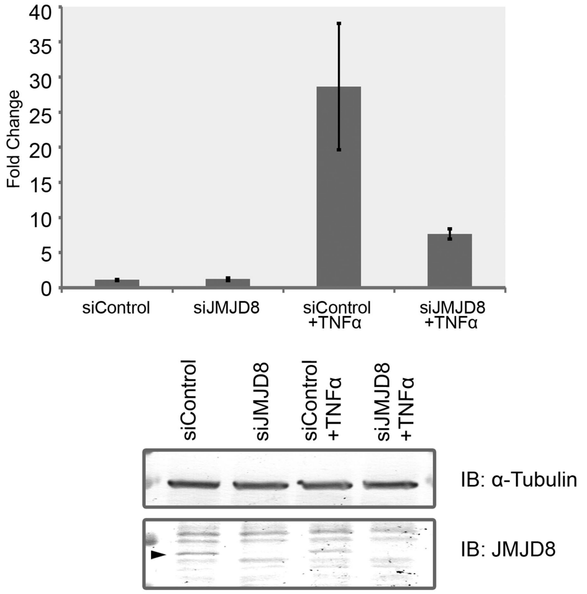

(b)

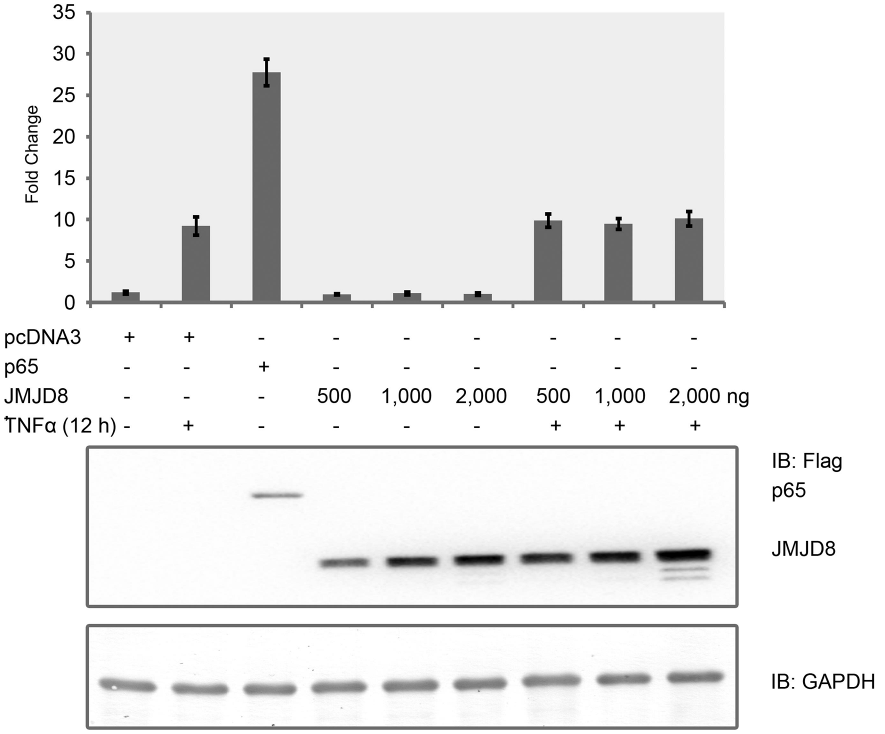

Supplementary Figure S1: JMJD8 is required for TNF-induced NF-κB activity. (a) Control and JMJD8 knockdown 293T-Luc cells were treated with 10 ng/ml of TNFα for 12 hours. Total cell lysates were prepared and luciferase activity was quantified with TECAN M200 microplate reader. (b) 293T-luc cells were transiently transfected with different construct as indicated and were treated with TNFα for 12 hours. Total cell lysates were prepared and luciferase activity was quantified with TECAN M200 microplate reader. The transient expression of ectopic JMJD8 was verified by immunobloting with the indicated antibodies. Data represent means ± SD. (n = 4).

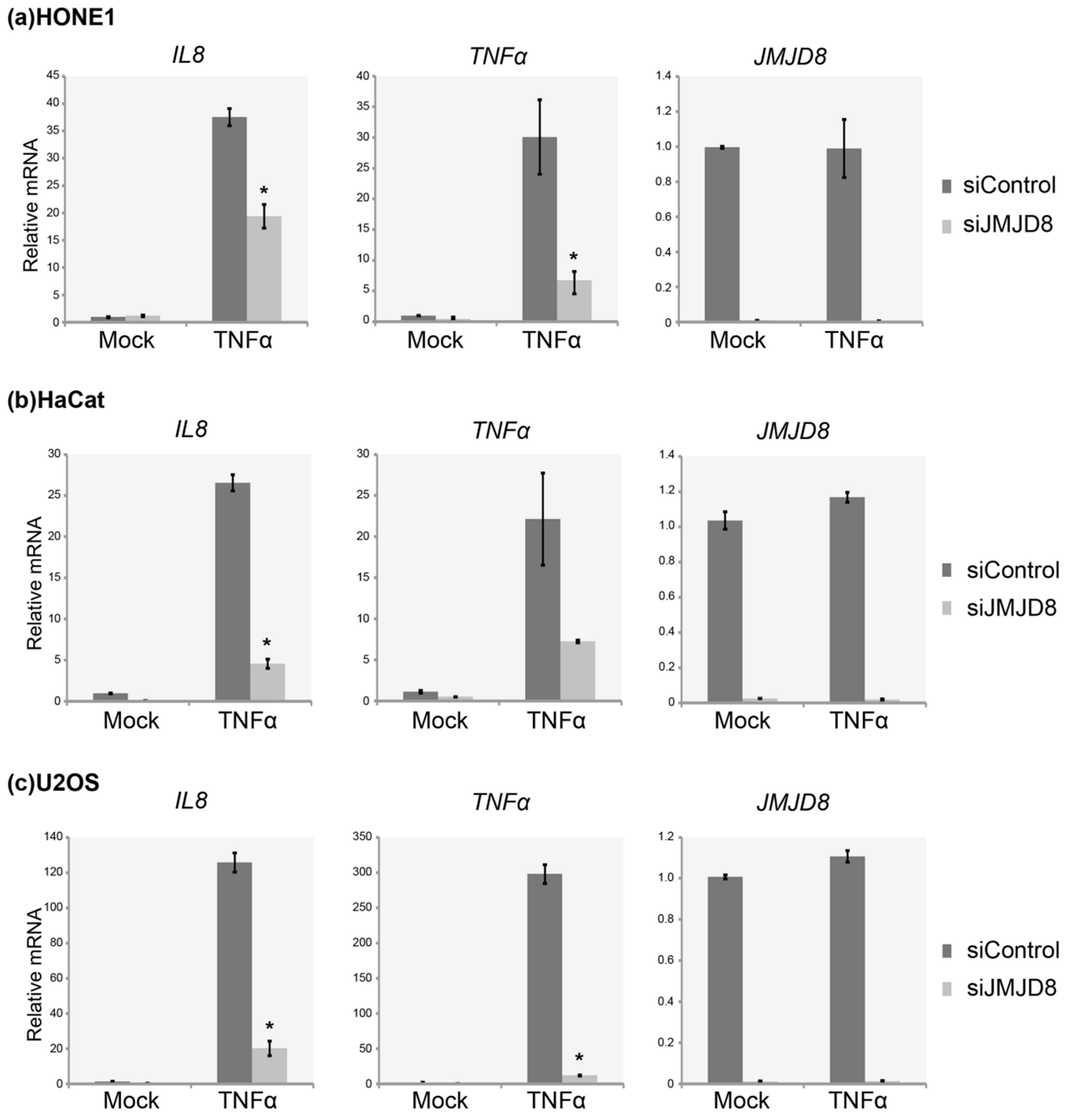

Supplementary Figure S2: JMJD8 regulates TNF-induced NF-κB signaling is not cell-type specific. (a) HONE1, (b) HaCat and (c) U2OS cells transfected with control and JMJD8 siRNA oligos were treated with or without 10 ng/ml of TNFα for 2 hours, the expression of *TNFα* and *IL8* were measured by RT-qPCR. (n = 4). Data represent means ± SD. (*p > 0.05).

(a)

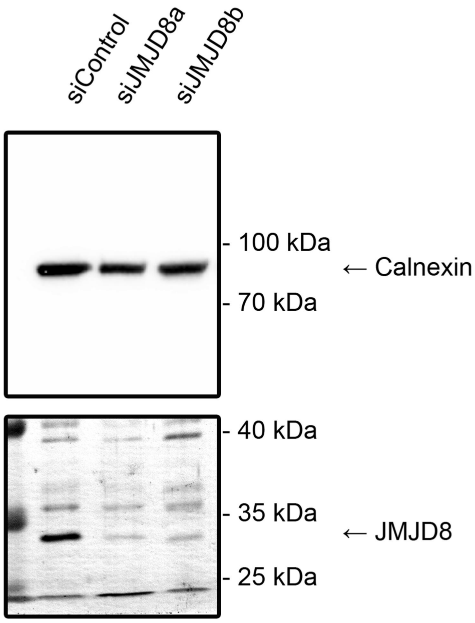

(b)

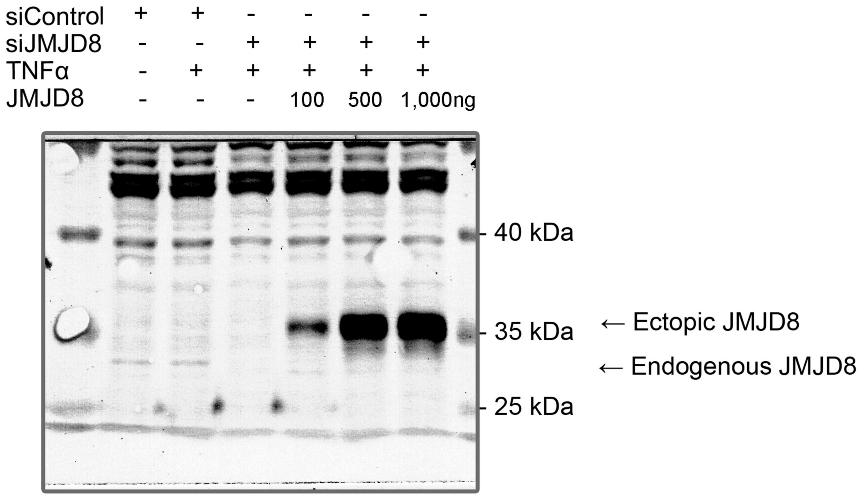

Supplementary Figure S3: Full-length images of immunoblots shown in Figure 1.

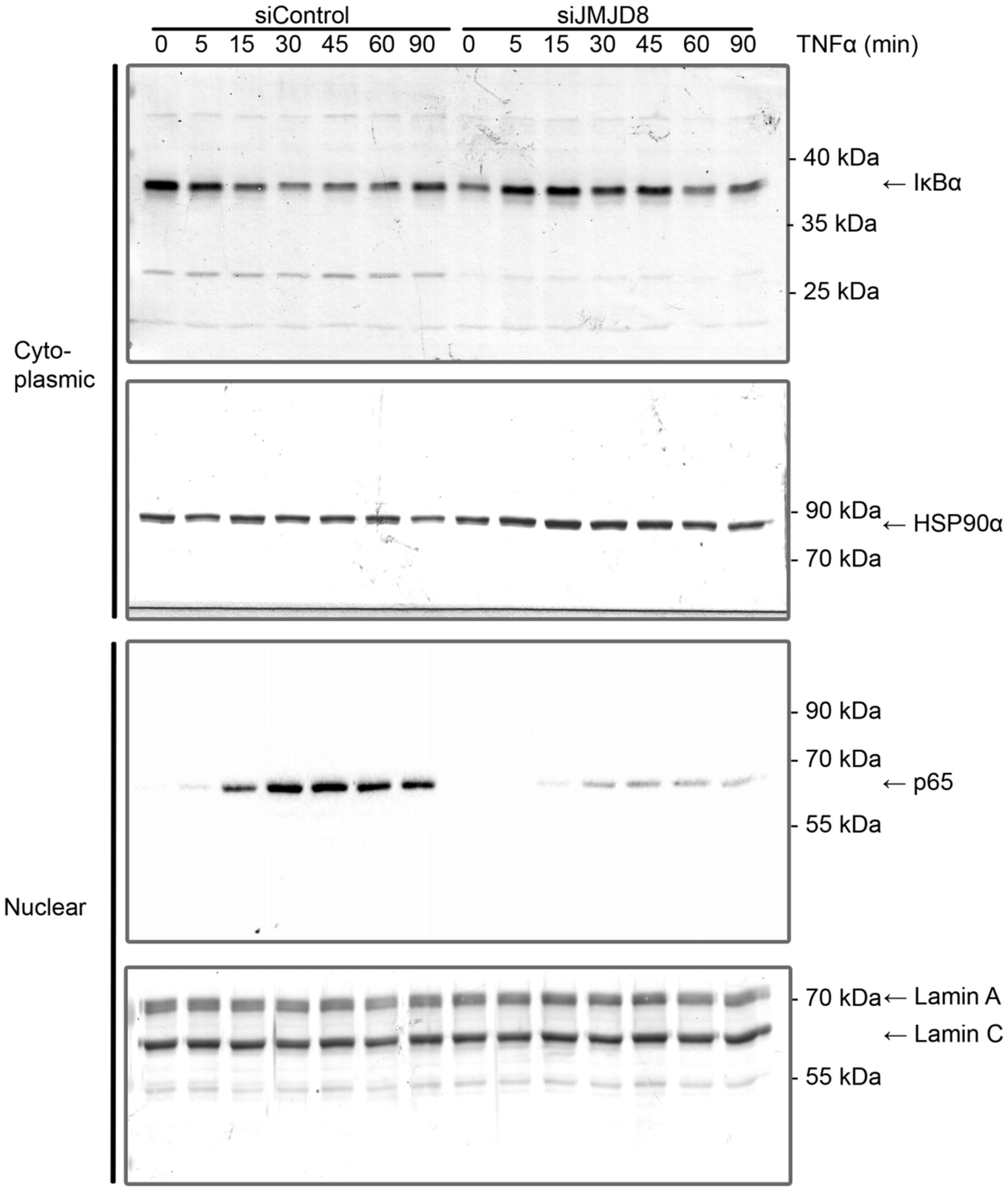

Supplementary Figure S4: Full-length images of immunoblots shown in Figure 2.

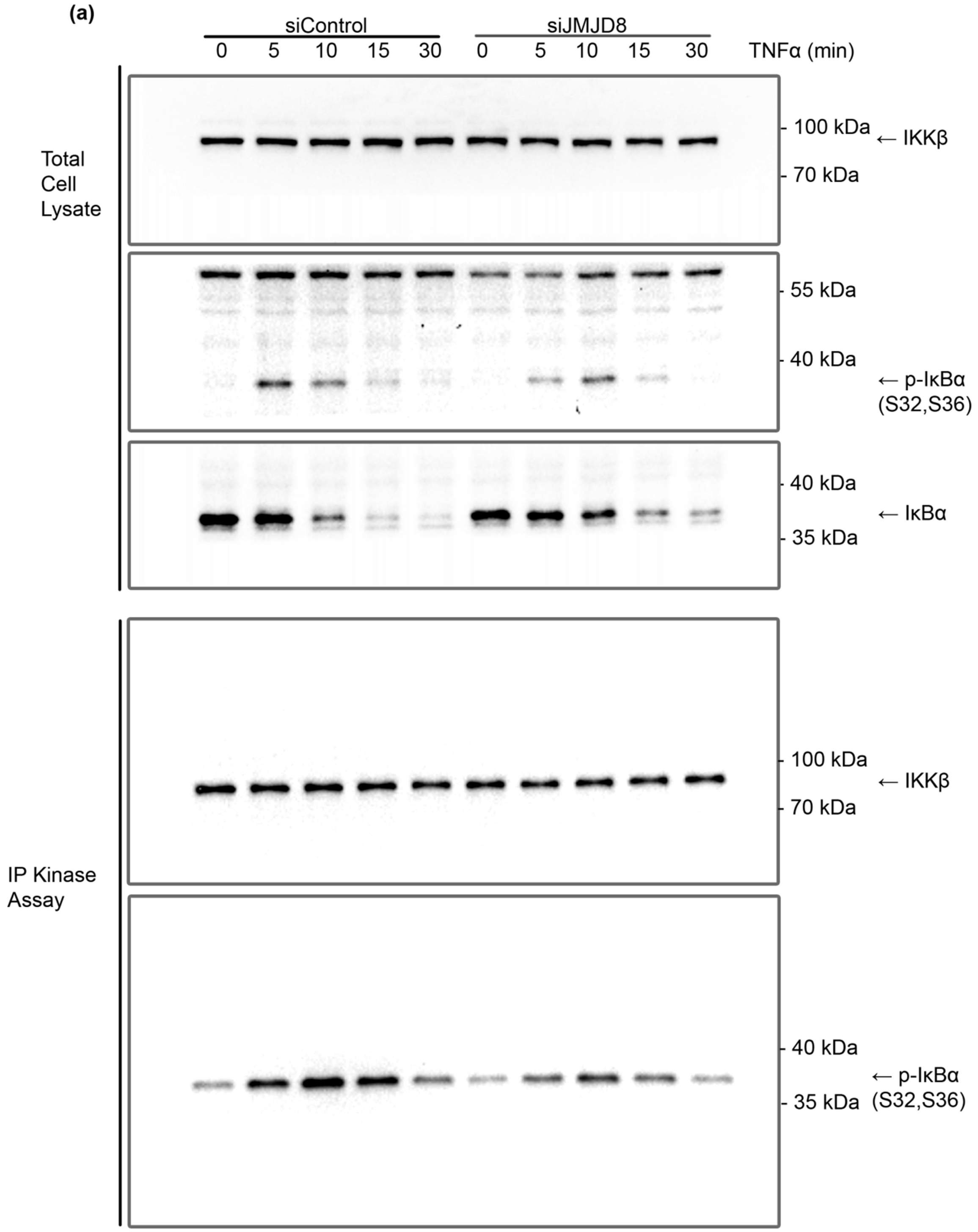

Supplementary Figure S5: Full-length images of immunoblots shown in Figure 3a.

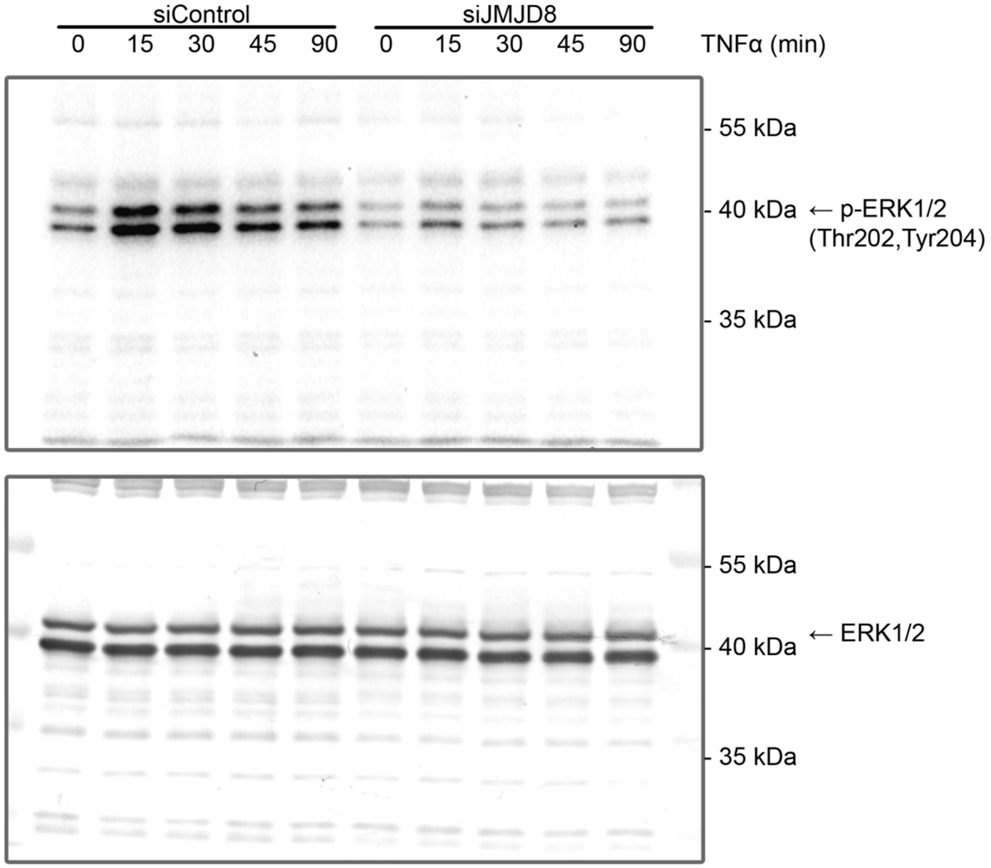

Supplementary Figure S6: Full-length images of immunoblots shown in Figure 3b.

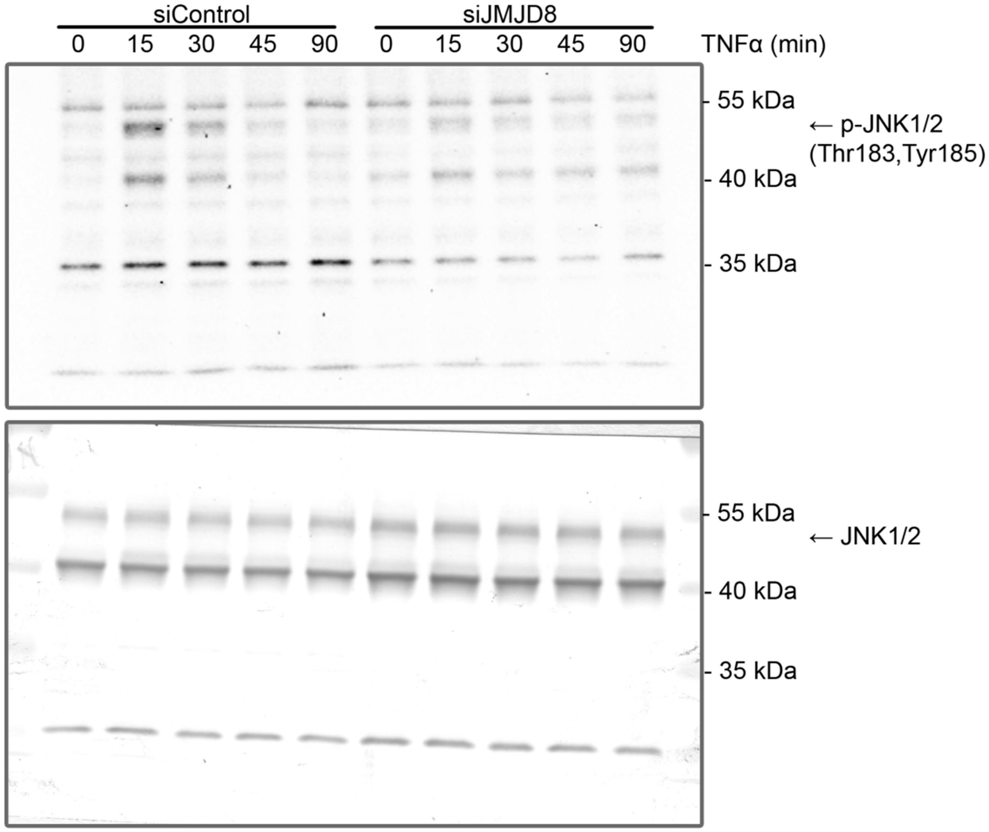

Supplementary Figure S7: Full-length images of immunoblots shown in Figure 3b.

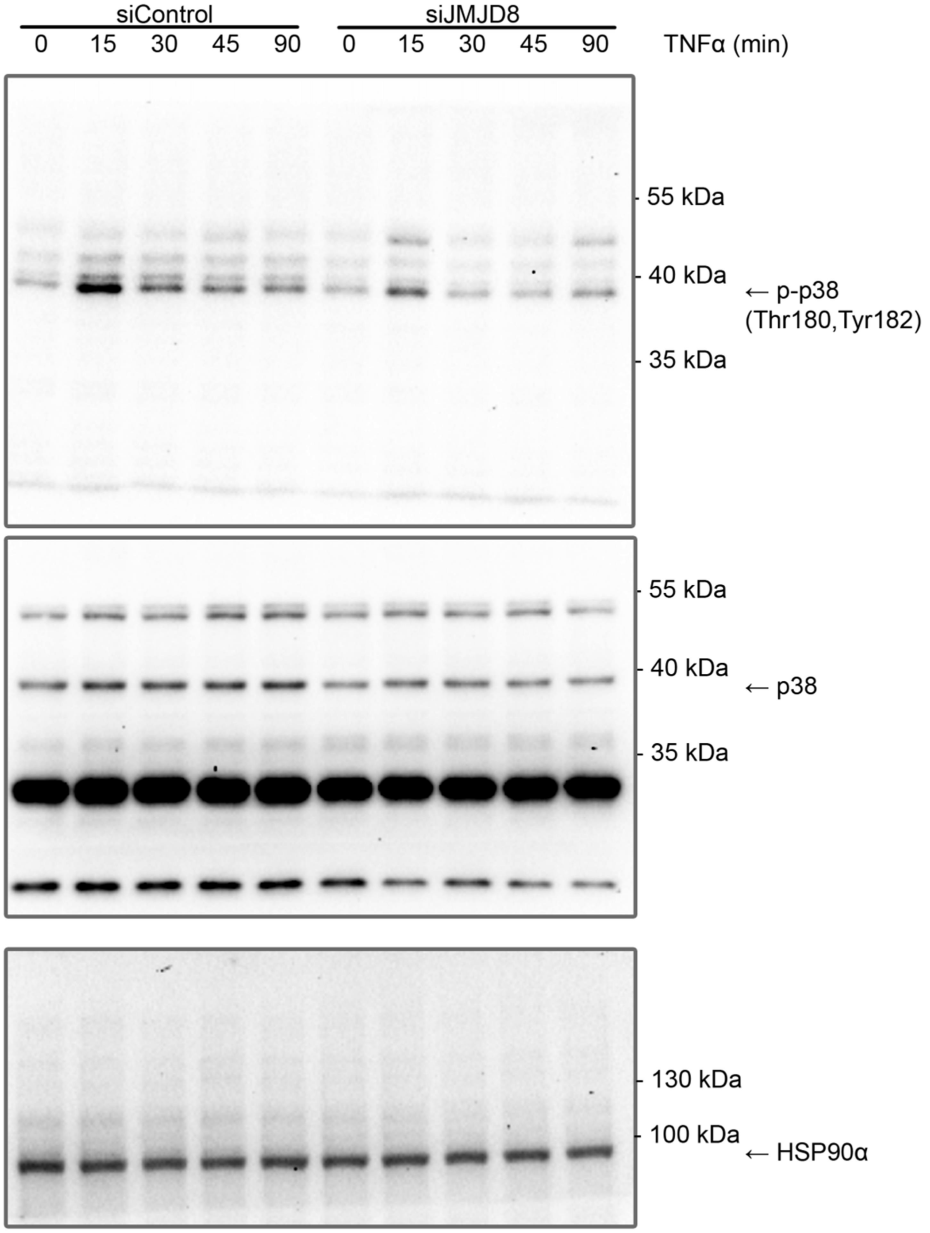

Supplementary Figure S8: Full-length images of immunoblots shown in Figure 3b.

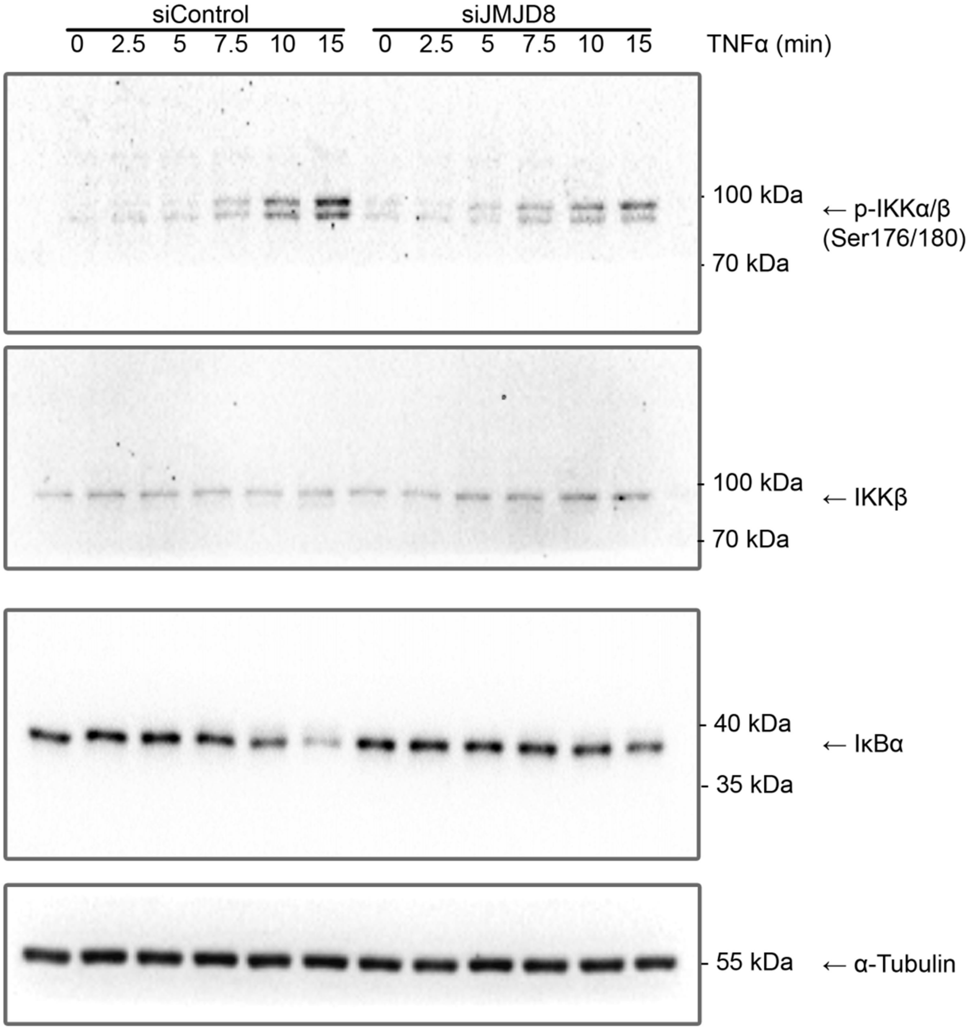

Supplementary Figure S9: Full-length images of immunoblots shown in Figure 4a.

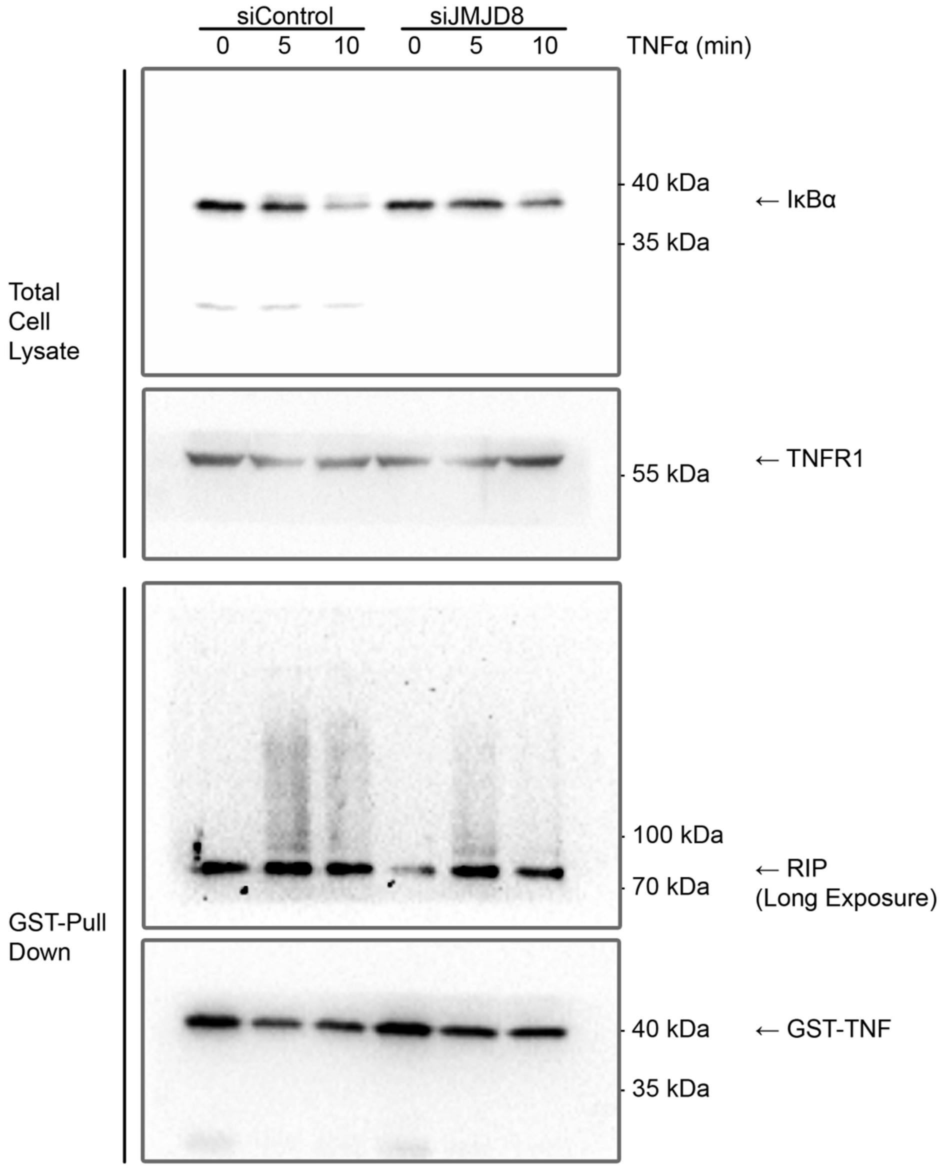

Supplementary Figure S10: Full-length images of immunoblots shown in Figure 4b.

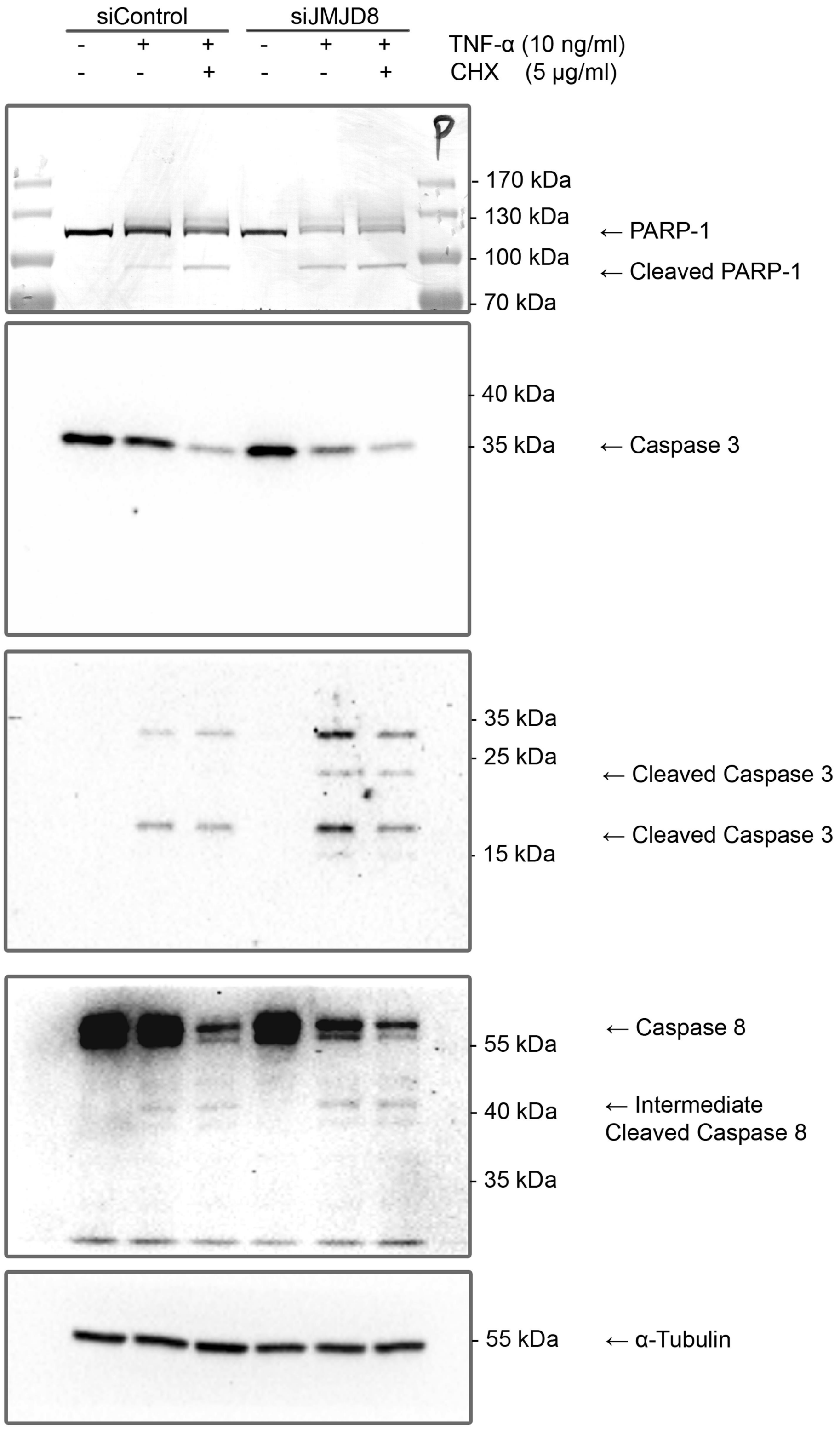

Supplementary Figure S11: Full-length images of immunoblots shown in Figure 5.

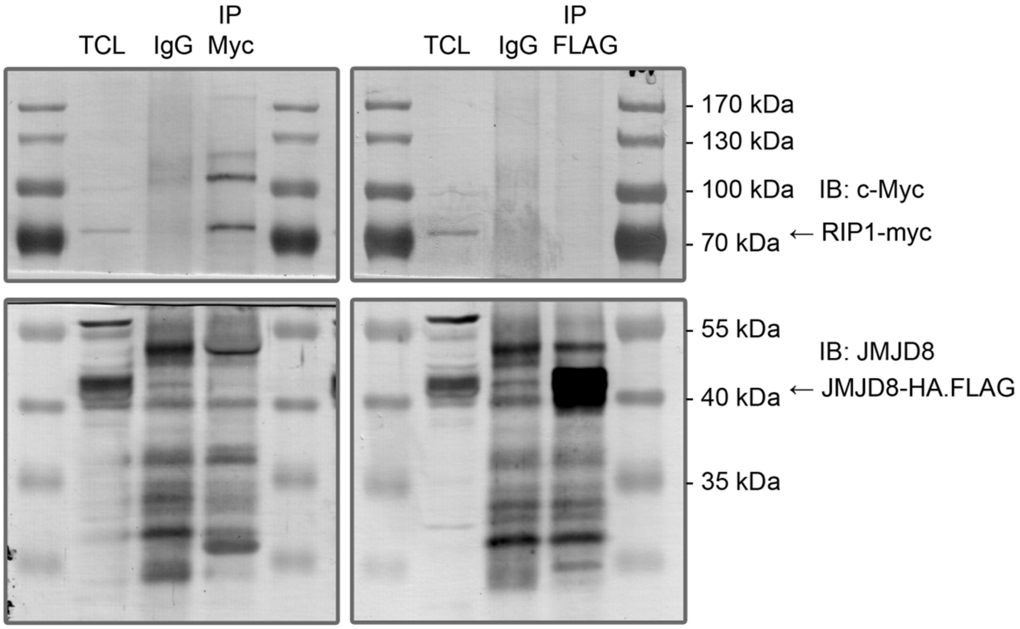

Supplementary Figure S12: JMJD8 does not interact with RIP1. HEK293T cells were transfected with RIP1-myc and JMJD8-HA-FLAG-expressing constructs. Total cell lysates were prepared 24 hours post-transfection and immunoprecipitated with the indicated antibodies. The bound proteins were analyzed by immunobloting with the indicated antibodies. TCL=Total cell lysate. (n = 2).
